# Effects of insulin and the glucagon‐like peptide 1 receptor agonist liraglutide on the kidney proteome in db/db mice

**DOI:** 10.14814/phy2.13187

**Published:** 2017-03-22

**Authors:** Leena Liljedahl, Jenny Norlin, James N. McGuire, Peter James

**Affiliations:** ^1^ Department of ImmunotechnologyLund UniversityLundSweden; ^2^ Novo Nordisk A/SMåløvDenmark

**Keywords:** GLP‐1R agonist liraglutide, insulin, kidney proteome, OPLS‐DA

## Abstract

Diabetes mellitus (DM) is a worldwide disease that affects 9% of the adult world population and type 2 DM accounts for 90% of those. A common consequence of DM is kidney complications, which could lead to kidney failure. We studied the potential effects of treatment with insulin and the glucagon‐like peptide 1 receptor (GLP‐1R) agonist liraglutide on the diabetic kidney proteome through the use of the db/db mouse model system and mass spectrometry (MS). Multivariate analyses revealed distinct effects of insulin and liraglutide on the db/db kidney proteome, which was seen on the protein levels of, for example, pterin‐4 *α*‐carbinolamine dehydratase/dimerization cofactor of hepatocyte nuclear factor‐1*α* (PCBD1), neural precursor cell expressed developmentally down‐regulated‐8 (NEDD8), transcription elongation factor‐B polypeptide‐1 (ELOC) and hepcidin (HEPC). Furthermore, the separation of the insulin, liraglutide and vehicle db/db mouse groups in multivariate analyses was not mainly related to the albumin excretion rate (AER) or the level of glycated hemoglobin A_1c_ (HbA_1c_%) in the mice. In summary, we show that insulin and liraglutide give rise to separate protein profiles in the db/db mouse kidney.

## Introduction

The prevalence of type 2 Diabetes mellitus (DM) is increasing rapidly (World Health Organization, 2014) with the number of people suffering from late complications of diabetes. One such late complication is diabetic kidney disease (DKD), leading to diabetic nephropathy (DN). It is the primary cause in the western world for end stage renal disease (ESRD), where the diabetic patient will need either dialysis or a kidney transplant in order to survive (Fabrizi et al. 2001; USRDS, 2015). Ultra‐structural tissue damage on the molecular level involves thickening of the tubular and glomerular basement membranes, extracellular matrix expansion and hypertrophy together with increasing albumin excretion rate (AER) and decreased glomerular filtration rate (Tervaert et al. 2010).

In the kidney, insulin receptors (IR) and insulin‐like growth factor‐1 receptors (IGF‐1R) are located mainly in the tubules and the glomeruli (Butlen et al. 1988; Uhlen et al. 2015) while in rodents and primates, GLP‐1 receptors (GLP‐1R) are located in the smooth muscle cells in the vascular walls (Pyke et al. 2014; Jensen et al. 2015). These receptors mediate important functions in the kidneys, as deletion of the IR in the proximal tubules has been shown to lead to increased hyperglycemia involving gluconeogenesis (Tiwari et al. 2013). Furthermore, transgenic mice with podocyte‐specific lack of IR has been shown to develop albuminuria (Welsh et al. 2010).

Liraglutide exhibited renoprotective properties in streptozotocin‐induced diabetic rats by reducing albuminuria and oxidative stress (Hendarto et al. 2012; Zhou et al. 2014) and recombinant GLP‐1 displayed natriuretic and diuretic effects in rat kidneys due to altered Na^+^ reabsorption in the tubules (Moreno et al. 2002). Thus, insulin, native GLP‐1 and liraglutide have an effect on kidney mechanisms; however, proteome differences in a diabetic kidney following insulin or liraglutide administration are not very well characterized. Mass spectrometry (MS)‐based proteomics is an important tool in determining mechanisms of action and responses to different treatments, combining high resolution with a theoretically unbiased protein detection approach (Mikami et al. 2012; Zhao and Lin 2014).

The leptin receptor‐deficient db/db mouse is one of the most widely used rodent models in type 2 DM research and it captures most of the early functional and structural changes seen in early DKD in humans. This includes kidney hypertrophy, elevated glomerular filtration rate, leakage of albumin into the urine (albuminuria), thickening of the glomerular basement membrane and mesangial expansion (Sharma et al. 2003; Breyer et al. 2005).

This study employs label‐free quantitative MS to study the effect of insulin and liraglutide on protein abundance levels in the kidney of db/db mice. Multivariate analyses were applied to investigate the dependence of the change in protein abundance between db/db mouse groups on such parameters as blood glucose (BG), HbA_1c_(%) and albuminuria.

## Materials and Methods

### Animals

The handling and use of all animals in this study was approved by The Danish Animal Experiments Inspectorate and was carried out according to the guidelines of The Council of Europe Convention for the protection of vertebrate animals used for experimental and other scientific purposes. Fifty‐eight 8‐week‐old male db/db and 14 db/+ mice from Charles River, Germany were housed five per cage in the animal unit facility at Novo Nordisk, Denmark in controlled temperature (23°C–24°C) with a 12‐h light/dark cycle. The animals were fed Altromin 1324 and tap water ad libitum upon arrival. After 2 weeks of acclimatization the db/db mice were divided into three dosing groups: vehicle control *n* = 18, liraglutide *n* = 20, and insulin glargine *n* = 20. The db/+ were used as nondiabetic vehicle controls (healthy control). Only db/db mice with BG above 16 mmol/L were included in the experiment. Vehicle dosing was done as previously described (Liljedahl et al. 2016). The dose of liraglutide (Novo Nordisk, Maaloev, Denmark) was escalated over 3 days with a first dose of 0.33 mg/kg, second dose of 0.66 mg/kg and the final dose of 1 mg/kg from day three until the termination. The first dose of insulin was 2 LinBits per 20 g mouse plus 1 LinBit (LinShin Canada Inc, Scarborough, ON, Canada) per additional 5 g of mouse. Thereafter, insulin glargine (Nomeco A/S, Copenhagen, Denmark) was given at a dose of 10 U/kg twice daily to adjust BG values to similar levels as in the liraglutide group. The animals were weighed weekly on a digital scale and doses were adjusted to current body weight. For the MS experiments, the left kidney was used from five mice per group with two technical replicates per kidney. The db/db mice given liraglutide (db/db liraglutide, *n* = 5) or insulin (db/db insulin, *n* = 5) were compared to a db/db vehicle group (db/db vehicle, *n* = 5) and to a group of db/+ vehicle mice (healthy control). Regarding the in vivo measurements, BG, HbA_1c_(%) and AER were assessed as previously described (Liljedahl et al. 2016).

### Protein purification and sample preparation

All solvents used for HPLC including formic acid (FA) and acetonitrile (ACN) (percentages are reported as (v/v)), ammonium bicarbonate, ammonium acetate, sodium chloride, sodium sulfite, 2,2,2‐trifluoroethanol (TFE), dithiothreitol (DTT), N‐acetyl‐cysteine and iodoacetamide (IAA) were purchased from Sigma‐Aldrich (Stockholm, Sweden). Zirconia beads were from BioSpec Products, Inc. (Bartlesville, OK) and sequencing grade modified trypsin was purchased from Promega (Madison, WI). The hydrazide Affi‐Prep resin (HZ) (BioRad, Hercules, CA) was supplied as a 50% slurry in isopropanol, sodium meta‐periodate was from Pierce (SDS diagnostics, Falkenberg, Sweden) and Sep‐Pak C_18_ columns were from Waters (Milford, MA).

100 mg of snap‐frozen kidney tissue was transferred to a Denator Stabilizor T1 (Svensson et al. 2009) (Denator, Gothenburg, Sweden) for inactivation of enzymatic processes. The tissue was homogenized using a bead beater with Zirconia beads for 4*1 min and kept on ice in between. Thereafter, the homogenized tissue from each kidney was divided into two samples; each sample was lysed in 0.5 mL 50% TFE/50% PBS, then 250 *μ*L 100 mmol/L ammonium bicarbonate at pH 8 was added and the samples were centrifuged at 4°C and 2000*g* for 30 min. The supernatant was incubated for 2 h at 60°C, samples were centrifuged at 2000*g* for 10 min at room temperature (RT) and then the supernatant was reduced with 5 mmol/L DTT at 60°C, shaking at 800 revolutions per minute (rpm) for 30 min, alkylated with 25 mmol/L IAA at RT in dark for 30 min followed by quenching with 30 mmol/L N‐acetyl‐cysteine for 15 min at RT in dark, all at pH 8. Samples were digested with 20 *μ*g trypsin per sample shaking at 1000 rpm and 37°C overnight. The digest was cleaned by reverse‐phase (500 mg) C_18_ chromatography. Peptides were eluted with 0.1% FA/50% ACN and the flow‐through was reduced on a SpeedVac (ThermoScientific, Waltham, MA) to 100 *μ*L final volume. In order to reduce the complexity of the samples before MS analysis, all samples were separated into two fractions; one containing glycosylated peptides, captured on hydrazide beads (investigated in a separate study (Liljedahl et al. 2016)) and the nonglycosylated fraction studied here. The complete sample was oxidized in 20 mmol/L sodium acetate/100 mmol/L sodium chloride at pH 5 with sodium meta‐periodate added to a 8 mmol/L concentration and incubated at 6°C at 600 rpm in the dark for 60 min. After termination with 30 mmol/L sodium sulfite in the dark for 15 min at RT, the samples were coupled at RT overnight to 0.5 mL hydrazide Affi‐gel in a 50% slurry of 100 mmol/L sodium acetate/1 mol/L sodium chloride at pH 4.5 and 37°C with gentle vertical agitation. The supernatant (non‐glycosylated peptide fraction) was collected and combined with the first wash of the HZ resin with 80% ACN, thereafter reduced on a SpeedVac, cleaned up by reverse‐phase C_18_ chromatography and dried on the SpeedVac.

### Mass spectrometry

The mobile phase A consisted of water/0.1% FA and the mobile phase B of ACN/0.1% FA. Samples were dissolved in 5% ACN and 0.1% FA for MS analyses. The shotgun MS of the kidney peptides was done on a Thermo linear trap quadrupole (LTQ) Orbitrap XL mass spectrometer (Thermo Electron, Bremen, Germany) as previously described (Kurbasic et al. 2015; Liljedahl et al. 2016). Peptides were eluted from the analytical column using a linear gradient of mobile phase B developed from 3%−35% B during 90 min. The shotgun MS proteomics data have been deposited to the ProteomeXchange Consortium (Vizcaíno et al. 2014) via the PRIDE partner repository (Vizcaíno et al. 2013) with the dataset identifier PXD002988 and 10.6019/PXD002988. A guide to the sample names is found in Table S1. The quantitative confirmation of the peptide levels was done using selected reaction monitoring (SRM) LC MS/MS analysis on an Eksigent 2D NanoLC system (Eksigent technologies) interfaced with a triple stage quadrupole (TSQ) Vantage mass spectrometer (Thermo Scientific, San Jose, CA). The separation system consisted of a trapping column (5 mm × 0.3 mm i.d, PepMap Acclaim C18, LC Packings, Sunnyvale, CA) and the analytical column was packed in‐house in 0.075 mm i.d PicoFrit (New Objective, Woburn, MA) with 3 *μ*m ReproSil C18‐AQ particles (Dr. Maisch, Ammerbuch, Germany) to a length of 15 cm. The LC gradient was run at 300 nL/min and consisted of a linear gradient of mobile phase B from 3% to 15% B during 3 min, from 15% to 35% B during 34 min, from 35% to 60% B during 30 min, from 60% to 90% B during 3 min followed by 10 min with 90% B. An injection for washing and equilibration of the column was performed between each sample. The TSQ MS was equipped with a nano electrospray interface operated in positive ion mode with a spray voltage of 1.8 kV and an ion capillary temperature of 270°C. Both Q1 and Q3 were set to unit resolution (0.7 Da) with a 10 msec dwell time. Data were acquired using the Xcalibur software, version 2.1.0 Sp1 (Thermo Fischer Scientific). The SRM MS data have been deposited in PASSEL (Farrah et al. 2012) via Peptide Atlas and are available with the dataset identifier PASS00840.

### Data analysis of shotgun MS

The shotgun raw files were imported into Progenesis QI v. 1.0.5156.29278 (Nonlinear Dynamics, Waters) and Proteios (Häkkinen et al. 2009) version 2.17.0 (Lund, Sweden). In Progenesis, spectra were automatically aligned, manually inspected and adjusted if needed. Normalization of intensities was done automatically within the Progenesis workspace using a global scaling factor. The built‐in quality control in Progenesis indicated well‐aligned spectra and agreement between normalized samples. All detected features were exported to Mascot (MatrixScience, London, UK) version 2.4.1 and searched against Uniprot mouse 2013.05 with equal number of reversed sequences. The mass tolerance for the parent ion was set to 5 ppm and to 0.8 Da for the fragment ions. One missed protease cleavage was allowed. Cys carbamidomethylation was set as fixed modification and Met oxidation as variable modification. Peptides with *P* < 0.05 were imported into Progenesis and peptides with reverse sequences and Mascot score <25 were removed. Peptides were assembled into a total of 1390 proteins and protein quantification was done based on nonconflicting peptide intensities. Peptides and proteins were filtered with 5% false discovery rate (FDR). In Proteios, Mascot search parameters were as mentioned above and peptide and proteins were filtered with 5% FDR. A spectral library was exported for SRM assay construction. The peptide file with normalized intensities from Progenesis can be seen in Table S2 in addition to the raw data deposition. Peptide and protein data for the proteins reported in this study can be seen in Tables S3–S4.

### Multivariate data analyses

Multivariate analyses were done with unsupervised principal component analysis (PCA) and supervised orthogonal partial least square discriminant analysis (OPLS‐DA) using SIMCA v. 14 (Umetrics, Umeå, Sweden). By including parameters like HbA_1c_(%), BG, AER, body weight (BW) and kidney weight (KW) (final measuring time point) in the OPLS‐DA model as a *y*‐variable, an examination can be performed of whether the parameter drives the separation of the samples or not. In this study the four groups of mice (db/db insulin, db/db liraglutide, db/db vehicle and healthy control) were defined as separate classes in the OPLS‐DA analyses. The Variable Importance for the Projection (VIP) plots visualizes variables separating the classes (reflecting the latent structures), where levels >1 indicate influence on group separation (Xie et al. 2015). In the shared and unique structures (SUS) plot three groups are compared simultaneously based on their covariance and correlation (Wiklund 2008; Wiklund et al. 2008). The SUS plot was applied on the db/db insulin and db/db liraglutide groups in relation to the db/db vehicle mouse group (common control) and unique and shared structures were visualized.

In all multivariate analyses, the variables albumin and *β*‐globin were removed, since their intensities were identified to be an artifact from two of the samples. All the samples, including technical replicates, were examined individually and thereafter the two technical replicates were joined for the statistical analyses.

### Quantitative confirmation of shotgun MS data

Confirmation of selected protein levels detected in the shotgun MS data was done by SRM MS. The selection of peptides was based on the multivariate analysis; indication of differential abundance level in the SUS plot and levels above 1 in the VIP plot, indicative of importance for mouse group separation, were used as selection criteria. Peptides were checked against an in silico digestion of the complete UniprotKB mouse proteome 2014.06 in order to ensure unique peptides were selected. Synthetic peptides were from JPT (Berlin, Germany). Expression signatures from the shotgun data for proteins and the corresponding peptides used in the SRM assay were carefully compared. 296 peptides from 90 proteins were tested for their suitability for SRM assays using SRM software Skyline (MacLean et al. 2010) v. 2.6.0.7176 (MacCoss lab, Washington). In the final SRM assay, 54 representative peptides from 50 proteins were included with a minimum of four transitions per peptide. The transition list is shown in Table S5. Nine of the 50 proteins represented by 12 peptides were included in the assay as standards for normalization based on their stable abundance levels detected in the shotgun MS dataset. All nine proteins remained unchanged in the confirmatory dataset. Each technical replicate was run as a dilution curve with 3–6 concentrations. One sample was run in triplicate; at the beginning, middle and end of the series. This sample was used to calculate the CV% for the normalized values. Maximum CV was 8%.

Skyline was used for analysis and inspection of the raw SRM data of all technical replicates. The peak areas for all transitions of a peptide were integrated and summed in order to obtain the total peak area for each peptide. SRM raw data were normalized with NormFinder (Andersen et al. 2004) after evaluation of the optimal normalization method using the software Normalyzer 1.1.1 (Chawade et al. 2014). All technical replicates were merged for the statistical analysis.

### Functional relationships

All the proteins included in the confirmatory SRM dataset were entered into the protein association networks database STRING v. 10.0 (Snel et al. 2000; Szklarczyk et al. 2015) in order to assess possible functional interactions or other known or predicted relationships between them. It included several mitochondrial proteins but no major differences between insulin and liraglutide administration. The network and graphs of some of the included protein abundances can be seen in Table S6.

### Statistical analyses

One‐way ANOVA with Tukey *post hoc* test was performed on mouse parameters and protein intensities in GraphPad PRISM 6 (La Jolla, CA). Brown–Forsythe's test, conducted simultaneously in PRISM, evaluates differences in the intra group variance. Variables found significant in Brown–Forsythe's test were not further analyzed. Default settings were used in GraphPad PRISM calculations, with *P* < 0.05 considered significant. Graph images are reported with SEM (mouse parameters) or 95% CI (protein data), as indicated in the figure text. Mouse parameters in the text are reported as mean (SD).

## Results

### Mouse parameters

**Figure 1:**
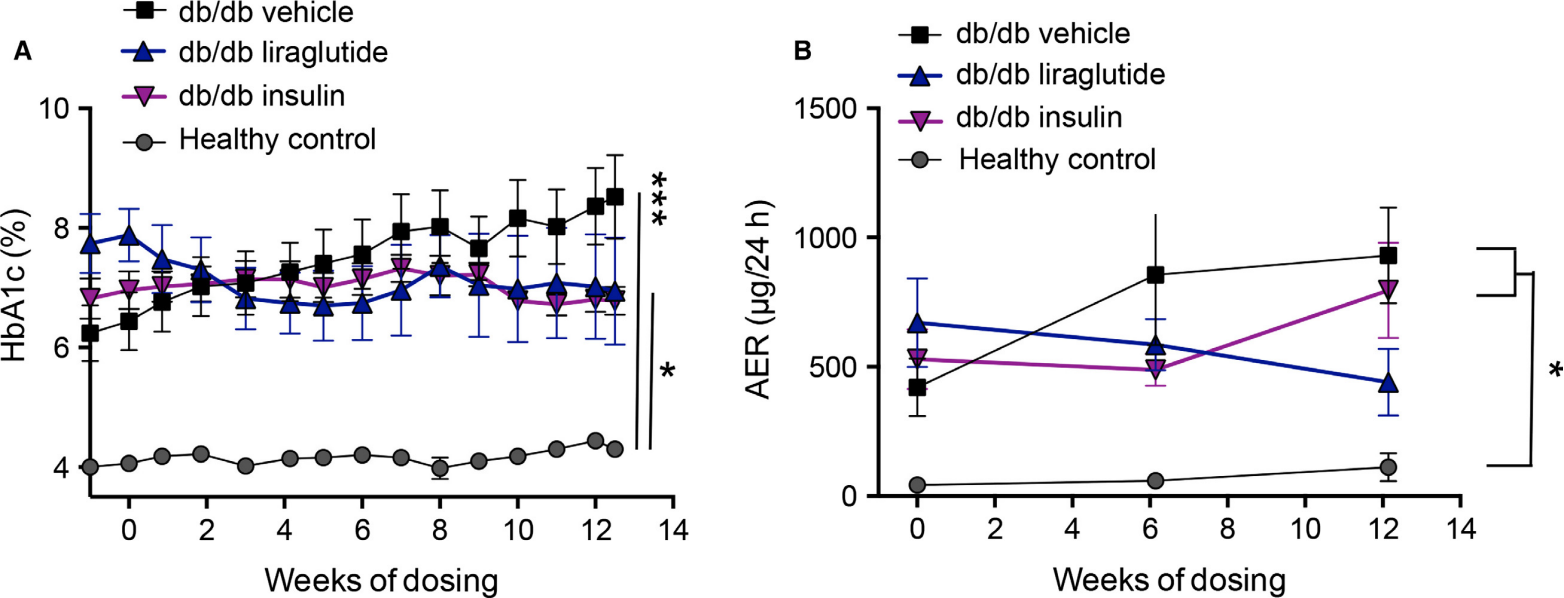
Mouse parameters HbA_1c_(%) and albumin excretion rate (AER). (A) The level of HbA_1c_(%) was significantly higher in the three db/db groups than in the healthy control group at all time points. At the terminal time point there was no significant difference within the three groups of db/db mice. The significance level at 12.5 weeks is indicated with asterisks in Panel (A). (B) After 12 weeks of dosing there was significant difference in the AER between the healthy control and db/db vehicle group (*P* = 0.013) and the healthy control and db/db insulin group (*P* = 0.041), indicated by an asterisk in the figure. Total *n* = 20, *n* = 5 in each group. One‐way ANOVA with Tukey *post hoc* test was performed, *P* < 0.05 was considered significant. *P < 0.05, **P < 0.01, ***P < 0.001. Data are presented as mean ± SEM.

After 12 weeks of vehicle, insulin or liraglutide dosing, HbA_1c_(%) (Fig. 1A) was 4.3 (0.2)% in the healthy control mice compared to higher levels in the three db/db mouse groups; db/db vehicle 8.5 (1.6)%, db/db insulin 6.8 (0.5)% and db/db liraglutide 6.9 (2.0)%. There was no significant difference within the three db/db groups after 12 weeks of dosing but HbA_1c_(%) was significantly increased in the three db/db groups compared to the healthy control mice (*P*‐values after 12 weeks of dosing; healthy control versus db/db vehicle *P* < 0.001, healthy control versus db/db liraglutide *P* < 0.026 and healthy control versus db/db insulin *P* < 0.038). The AER was analyzed at baseline, 6 and 12 weeks (Fig. 1B). At baseline there was no significant difference between the two vehicle groups at 43 (20) *μ*g/24 h (healthy control, *n* = 4) and 421 (250) *μ*g/24 h (db/db vehicle, *n* = 5) although the mean values were widely separated. The AER values in the db/db insulin mice (529 (257) *μ*g/24 h, *n* = 5) and in the db/db liraglutide mice (671 (297) *μ*g/24 h, *n* = 3) were increased compared to the healthy control mice. At 6 weeks the AER was significantly increased (*P* < 0.02) in the db/db vehicle mice (855 [608] *μ*g/24 h, *n* = 5) compared to the healthy control mice (59 [27] *μ*g/24 h, *n* = 4) while the mean AER was decreased in the db/db insulin mice (489 [140] *μ*g/24 h, *n* = 5) and in the db/db liraglutide mice (585 [199] *μ*g/24 h, *n* = 4) although no significant difference was detected involving these mouse groups. After 12 weeks of dosing the AER was significantly higher in the db/db vehicle mice (930 [413] *μ*g/24 h, *n* = 5, *P* < 0.014) and the db/db insulin mice (795 [411] *μ*g/24 h, *n* = 5, *P* < 0.042) compared to the healthy control (112 [109] *μ*g/24 h, *n* = 4). There was no significant difference between the db/db liraglutide mice (440 [256] *μ*g/24 h, *n* = 4) and any other mouse group (Fig. 1B).

### Effects of insulin and liraglutide on the proteome

**Figure 2:**
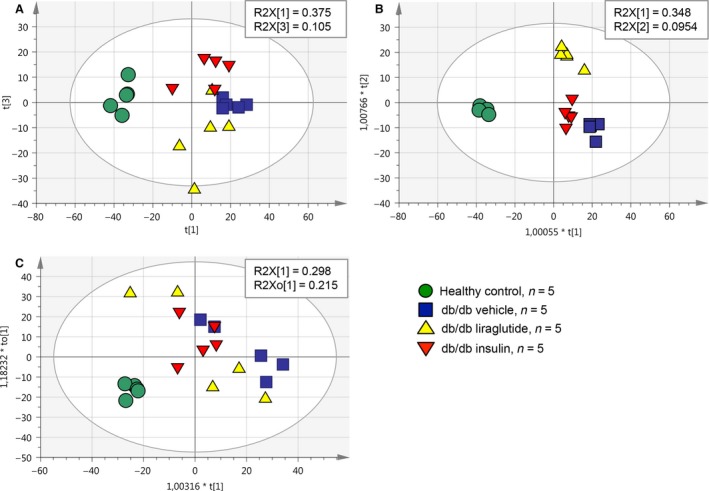
Multivariate data analyses of the shotgun data. The abundances of the 1390 proteins identified in the shotgun MS analysis were used as variables in the multivariate analyses. (A) Component 1 and 3 are shown in the PCA of the four mouse groups included in this study. The healthy control group was clearly separated from the three db/db mouse groups. Insulin and liraglutide had separate effects on the protein abundances in the db/db mouse model, although the three db/db mouse groups were not completely separated. Statistical parameters for the PCA were R2X(cum) = 0.615, Q2(cum) = 0.366 with three components (obtained from SIMCA). (B) Supervised OPLS analysis of the four mouse groups is shown where the db/db groups are separated from each other. Statistical parameters for the model are R2X(cum) = 0.794, R2Y(cum) = 0.957, Q2(cum) = 0.716 and 3+3+0 latent structures (obtained from SIMCA). (C) The level of HbA_1c_(%), BG, KW/BW and AER after 12 weeks of dosing were included, respectively, and together as *Y*‐variables in the OPLS analysis of the four mouse groups. In C HbA_1c_(%) is shown as an example of an included mouse parameter, illustrating that when including these *y*‐variables, the three db/db mouse groups were not well separated from each other. SIMCA statistical parameters for the model were R2X(cum) = 0.364, R2Y(cum) = 0.737 Q2(cum) = 0.639 and 1+1+0 latent structures.

The majority of the protein differences in the shotgun MS dataset were mouse model specific as seen in the PCA illustrated in Figure 2A. The healthy control group was readily separated from the three groups of db/db mice, which seemed to overlap slightly, as illustrated in Figure 2A with the principal components 1 and 3. For enhanced examination of the differences between the three db/db groups, supervised OPLS analysis was applied as illustrated in Figure 2B. In the OPLS analysis, all four mouse groups were separated with good statistics for the model (shown in Fig. 2), although the major separation still was seen between the healthy control and the three db/db mouse groups. When mouse parameters (BG, HbA_1c_(%), BW, KW and AER) were included in the OPLS model, individually or together, there was no clear separation of the three db/db mouse groups, demonstrating that the separation of the db/db groups was not driven by those parameters (e.g., in Fig. 2C). The healthy control group was at all time points well separated from the three db/db mouse groups. In general the protein expression in the db/db mice was regulated similarly by both insulin and liraglutide, however, both the PCA and the VIP plots in the OPLS‐DA indicated that several proteins were uniquely regulated by the two treatments.

### Individual protein levels

**Figure 3:**
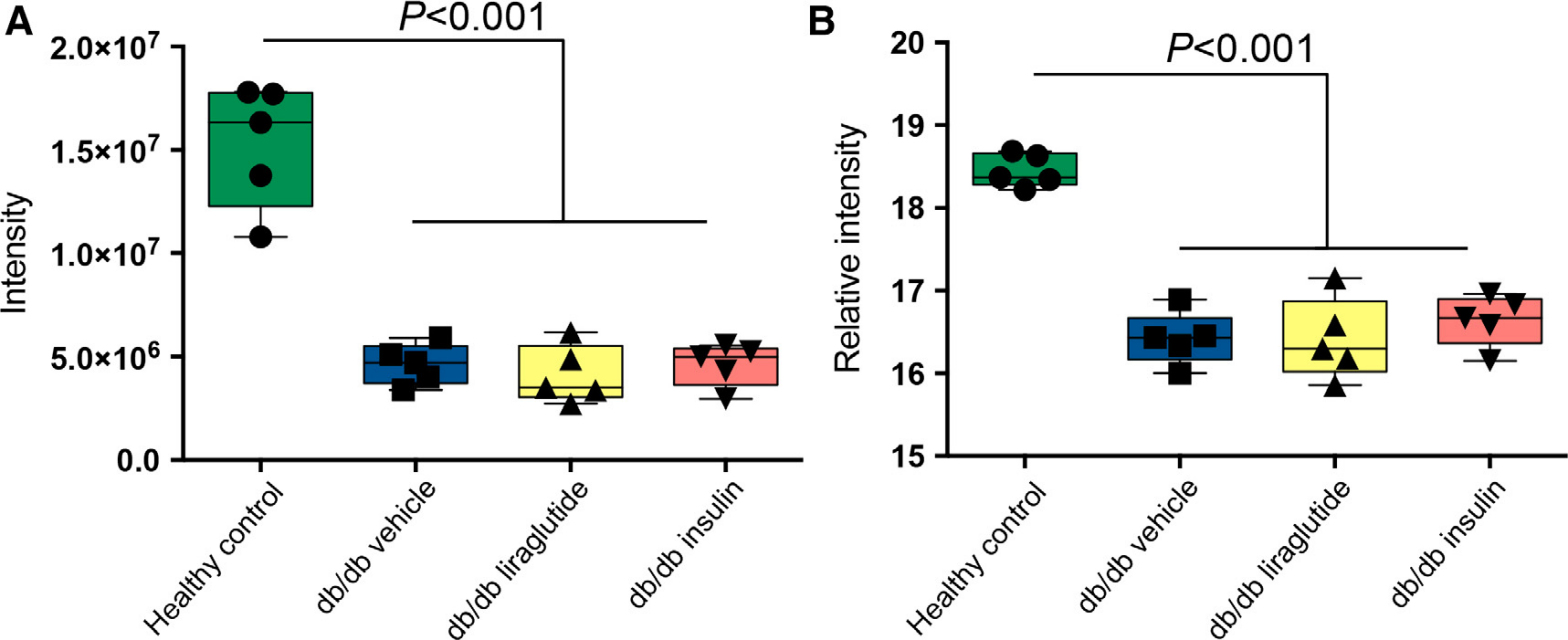
Quantitative confirmation of shotgun data with SRM. Both graphs show meprin‐*α* subunit‐*β* (MEP1B). Panel (A) shows shotgun MS and panel (B) targeted SRM MS. One‐way ANOVA and Tukey *post hoc* test was applied, *P* < 0.05 was considered significant. Mean and 95% confidence intervals are indicated in the figure.

**Figure 4:**
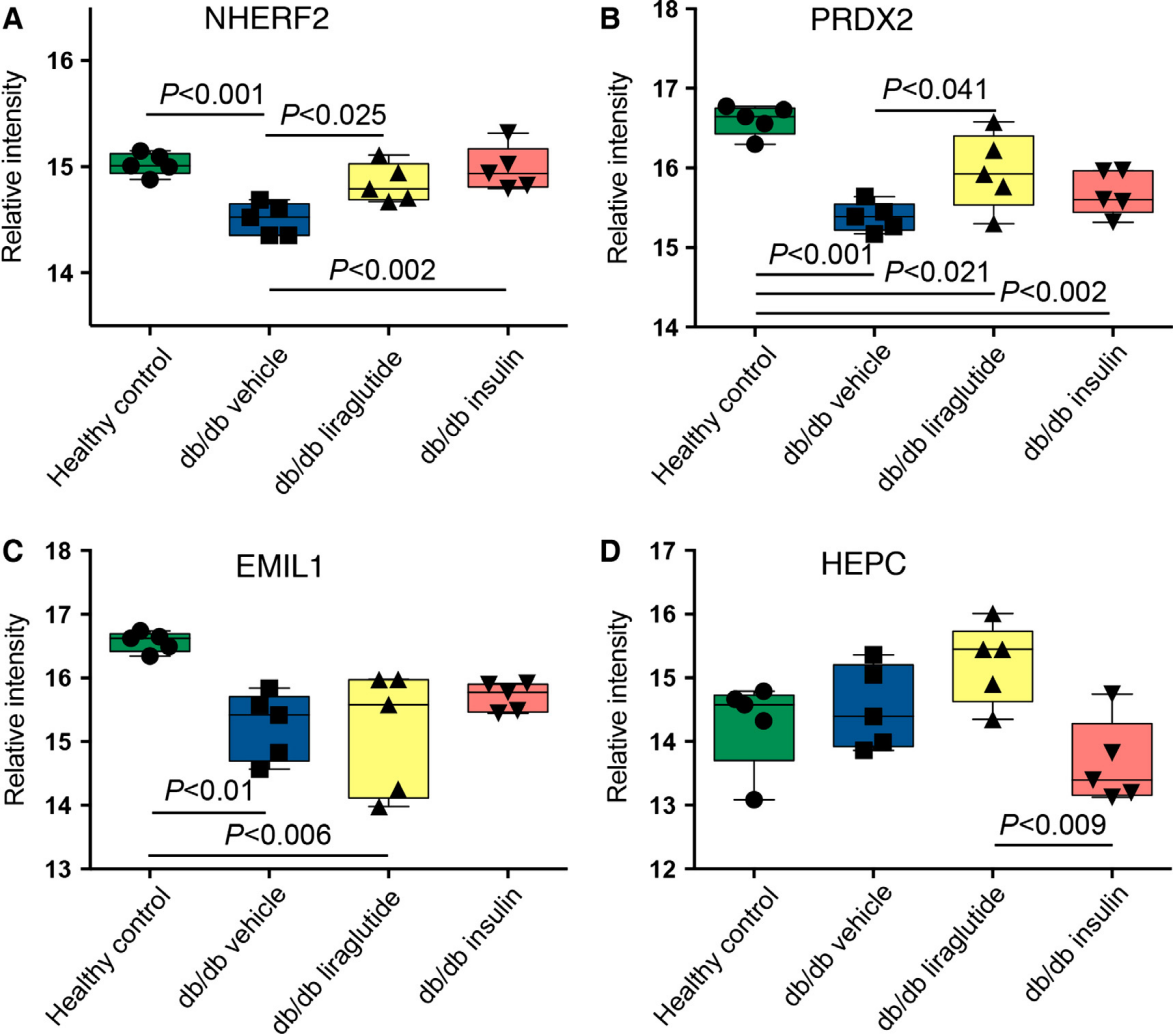
The effect of liraglutide or insulin on protein abundances. For the proteins illustrated in panel (A–D), it could be seen that insulin and liraglutide had both similar and opposite effects on the protein abundances. (A–D) show targeted SRM MS data with relative normalized abundances. One‐way ANOVA and Tukey *post hoc* test was applied to all groups and proteins, in graphs mean with 95% confidence interval are indicated. *P* < 0.05 was considered significant.

**Figure 5:**
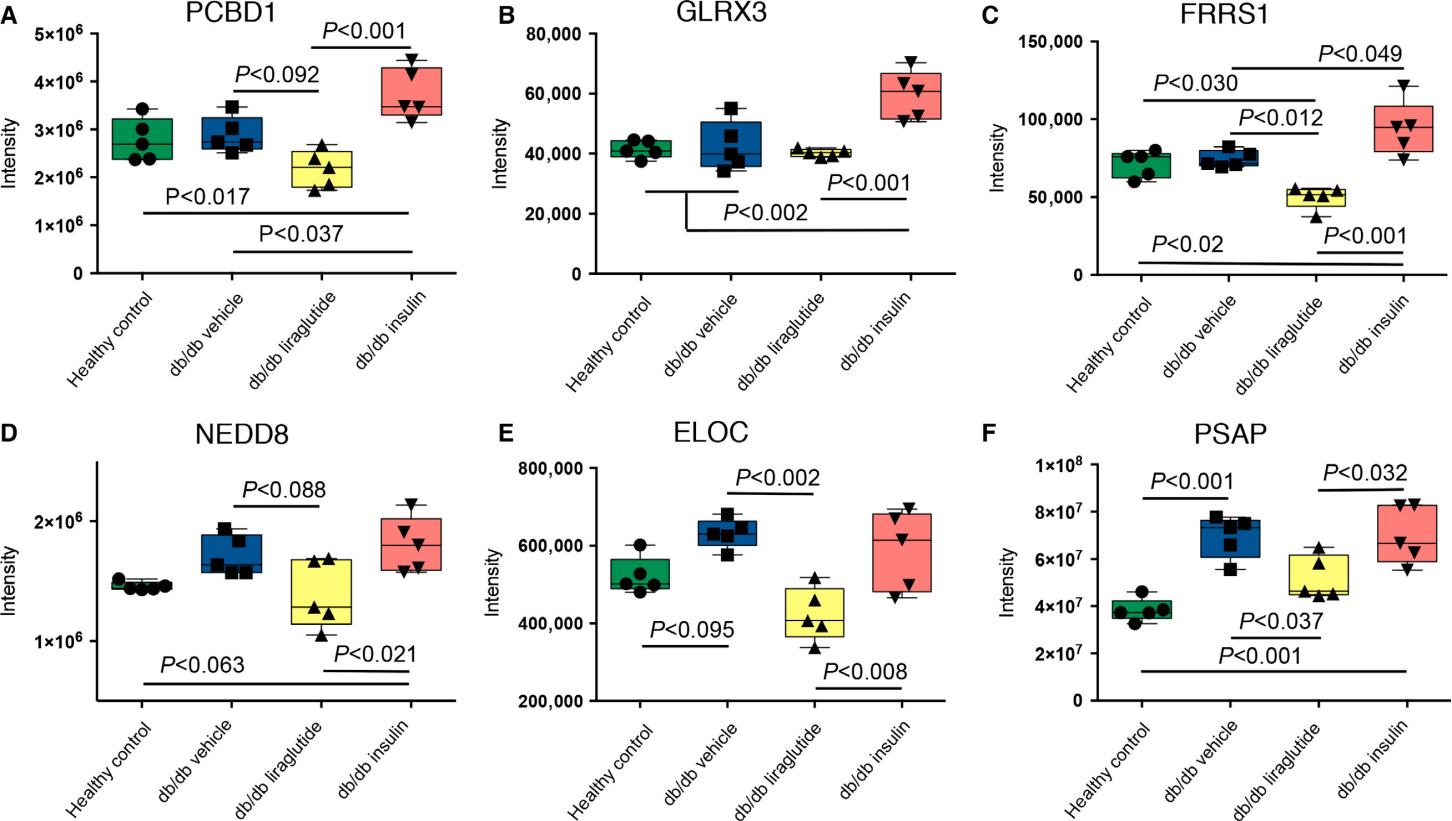
Shotgun data with insulin and/or liraglutide effects on protein abundances. Panel (A–F) show distinct effects of insulin and liraglutide on the protein abundance levels in the db/db mouse model. One‐way ANOVA and Tukey *post hoc* test was applied, *P* < 0.05 was considered significant. In graphs mean with 95% confidence interval are indicated. In PCBD1, NEDD8 and ELOC trends toward differential protein abundances (*P* < 0.1) are shown.

The univariate differences between the mouse groups, identified in the VIP and SUS plots, were analyzed using one‐way ANOVA. Selected protein abundance levels were confirmation using SRM MS. These proteins did not necessarily have significantly different abundance levels between the mouse groups in the shotgun data, since small differences also are included in the multivariate modeling. Shotgun and SRM abundance levels agreed well as illustrated in Figure 3. Sixteen of the proteins in the SRM assay had significantly different abundance levels between at least two of the four groups in the study. These proteins are listed in Table 1. In six of the proteins, the significant difference was between the healthy control group and the three db/db groups or between the two vehicle groups (db/db vehicle vs. healthy control). Examples of four of the proteins which abundances were confirmed with SRM are reported below and their relative intensities are shown in Figure 4 and Table S4. Both insulin and liraglutide increased the protein abundance of Na^+^H^+^ antiporter 3 regulatory cofactor‐2 (SLC9A3R2/NHERF2) in the db/db mice toward the level in the healthy control mice (Fig. 4A). The abundance of peroxiredoxin‐2 (PRDX2) was also significantly lower in the db/db vehicle compared to the healthy control mice and liraglutide significantly increased the protein abundance toward the level in the healthy control mice. Insulin had a small nonsignificant effect on the PRDX2 protein level, illustrated in Figure 4B. For emilin‐1 (EMIL1) the db/db vehicle and db/db liraglutide groups had significantly lower protein levels compared to the healthy control mice, illustrated in Figure 4C. Insulin had a small effect on the protein level and this group was not significantly different from any of the other mouse groups. The abundance of hepcidin (HEPC) was significantly increased in the db/db liraglutide compared to the db/db insulin group, illustrated in Figure 4D. In the multivariate analysis PCBD1 was identified as one of the proteins displaying differential regulation in the liraglutide and insulin mouse groups and it was included in the initial SRM assay, though no suitable peptide could be found. The abundance of PCBD1 was significantly higher in the insulin group compared to the other three groups of mice (Fig. 5A).

**Table 1:** Proteins with significant protein abundance levels

Uniprot accession	Protein name	Short name	Number of identified peptides	Mascot Score	bRegulation of db/db vs. db/+	Regulation within db/db; insulin or liraglutide vs. vehicle
Mouse model specific						
Q9CPU0	Glyoxalase‐1	GLO1^a^	8	256	up	ns
Q61847	Meprin‐*α* subunit‐*β*	MEP1B^a^	11	1087	down	ns
P16015	Carbonic anhydrase‐3	CAR3^a^	1	32	down	ns
P15947	Kallikrein‐1	KLK1^a^	2	125	down	ns
P16045	Galectin‐1	LGAL1^a^	5	511	down	ns
Q9WVM8	Kynurenine/*α*‐aminoadipate aminotransferase	AADAT^a^	4	353	down	ns
Only vehicle groups differ						
P05202	Aspartate aminotransferase	GOT2^a^	5	350	down	ns
P26350	Prothymosin‐*α*	PTMA^a^	2	677	up	ns
P54071	Isocitrate dehydrogenase‐2	IDH2^a^	9	548	down	ns
P14206	Laminin receptor‐1	LAMR1^a^	2	142	down	ns
Mouse model and liraglutide effect						
Q61171	Peroxiredoxin‐2	PRDX2^a^	3	162	down	liraglutide up
Liraglutide effect						
Q8CHT0	Delta‐1‐pyrroline‐5‐carboxylate dehydrogenase	ALDH4A1^a^	3	187	down	liraglutide up
P70441‐2	Na(+)/H(+) exchange regulatory cofactor‐1 (putative)	NHERF1/SLC9A3R1	16	2219	up	liraglutide down^c^
Q3TWL8	Prosaposin (putative)	PSAP	12	1079	up^c^	liraglutide down
P83940	Transcription elongation factor B polypeptide 1/Elongin C	ELOC	3	175	up	liraglutide down
Q3UI46	Neural precursor cell expressed developmentally down‐regulated‐8	NEDD8	3	162	ns	liraglutide down
Insulin effect						
P00920	Carbonic anhydrase‐2	CAR2^a^	6	408	down	insulin up^c^
Q99K41	Emilin‐1	EMIL1^a^	3	213	down	insulin up
Q9CQM9	Glutaredoxin‐3	GLRX3	1	50	ns	insulin up
Insulin and liraglutide effect						
Q9JHL1	Na(+)/H(+) exchange regulatory cofactor‐2	NHERF2^a^ SLC9A3R2	2	188	down	insulin and liraglutide up
Q9EQ21	Hepcidin	HEPC^a^	1	28	ns	insulin down, liraglutide up
P61458	Pterin‐4 *α*‐carbinolamine dehydratase/dimerisation cofactor of hepatocyte nuclear factor‐1‐*α*	PCBD1	4	256	ns	insulin up, liraglutide down
Q8K385	Ferric‐chelate reductase 1	FRRS1	1	40	ns	insulin up, liraglutide down

This Table is divided into sets describing the main regulation pattern for the protein abundances. The direction of the regulation between the db/db vehicle mice and the healthy control mice is shown in column 6 and the direction of the regulation by liraglutide and/or insulin compared to the db/db vehicle mice is shown in column 7.

a Protein abundances verified by SRM MS. The regulation up or down reported in the last two columns corresponds to the mean value in the one group compared to another group (db/db vehicle compared to healthy control; db/db insulin vs. db/db vehicle or db/db liraglutide vs. db/db vehicle).

b Regulation between vehicle groups only.

c Trends have *P*‐values <0.1, all other shown regulations are significant with *P* < 0.05 calculated with one‐way ANOVA and Tukey *post hoc* test for multiple comparison.

Insulin, liraglutide or both had and an impact on the abundances of glutaredoxin‐3 (GLRX3), ferric‐chelate reductase‐1 (FRRS1) and NEDD8, illustrated in Figure 5B–D. However, no difference was detected in the protein abundances between the healthy control and the db/db vehicle mouse groups. The protein abundances of ELOC and prosaposin (PSAP) were higher in the db/db vehicle mice compared to the healthy control mice. Liraglutide decreased the protein levels of PSAP toward the healthy control mice and of ELOC even beyond the levels in the healthy control mice. Insulin did not have an effect on the levels of ELOC and PSAP (Fig. 5E–F).

## Discussion

There are receptors for both insulin and GLP‐1 in the rodent kidney, but whether distinct proteome changes appear upon their activation is not fully known. Thus, we set out to uncover whether administration of insulin and a GLP‐1R agonist would give rise to different proteome profiles in the db/db mouse kidney. Using mulitvariate analysis, we detected distinct effects in the db/db mouse kidney proteome. Additionally, when we included mouse parameters like HbA_1c_(%) in the multivariate model, a separation of the three db/db mouse groups was not achieved. Hence, our results point in the direction that the protein changes we see in the db/db mouse kidney were not mainly an effect of the parameters BG, HbA_1c_(%), BW, KW or AER (e.g., shown in Fig. 2C), but could be an effect of a more local response to insulin and GLP‐1R activation in the mouse kidney. Direct action of a GLP‐1 analog on the kidneys has previously been described with the GLP‐1R agonist exendin‐4, which mediated anti‐inflammatory effects on rat kidneys by direct interaction with the GLP‐1R (Kodera et al. 2011). Direct effects of insulin signaling in the podocytes have also been shown to be of high importance for podocyte survival (Welsh et al. 2010).

In the multivariate analyses several of the proteins accounting for the differences between the four groups of mice were identified as mitochondrial proteins. The mitochondria holds a key function in diabetes due to its importance in metabolism and energy flux (Ramakrishna et al. 2001; Befroy et al. 2007; Ogata et al. 2014). We found that the protein abundance of PRDX2, a thioredoxin‐dependent peroxidase reductase, important for the cellular antioxidant defense involving hydrogen peroxide clearance, was significantly increased by liraglutide compared to the db/db vehicle mice, towards the protein level in the healthy vehicle mice. Downregulation of PRDX2 has been shown to increase podocyte death in rats, mediated by ANG II (Hsu et al. 2011).

An increased degree of inflammation is frequently seen in DN and in diabetes late complications in general (Astrup et al. 2008; Overgaard et al. 2013). Prothymosin (PTMA), involved in inflammation (Ioannou et al. 2013), was detected with the highest protein abundance level in the db/db vehicle mice. Meanwhile, the protein abundance of hepcidin, shown to mediate protection in mice from inflammation by modulation of the acute inflammatory response (Maliken et al. 2011) was increased in the db/db liraglutide mice in our data.

In addition to increased inflammation and oxidative stress, hypertension and thickening of the basement membrane are known features of DN. Here, the protein abundance of EMIL1, an extracellular matrix (ECM) glycoprotein, was increased in the db/db insulin mice. Opposite to several other ECM proteins which increase fibrosis, EMIL1 has been shown to be beneficial in the vascular wall since it inhibits the transforming growth factor‐*β* (Zacchigna et al. 2006), a protein known to increase hypertension and ECM abnormalities (Munjal et al. 2014).

As described for EML1, PRDX2, and HEPC, they were mainly affected by either liraglutide or insulin, compared to the db/db vehicle mice. Oppositely regulated protein abundances as a result of insulin or liraglutide administration were seen for a rather small group of proteins. One of them, the moonlighting protein PCBD1 is localized in the proximal and distal convoluted tubules (Résibois et al. 1999) in the kidneys. In this study, we found that insulin significantly increased the protein abundance of PCBD1, and liraglutide slightly reduced the PCBD1 abundance in the db/db mice compared to the db/db vehicle group. A recessive mutation in PCBD1 involving its binding and activity as a cofactor of hepatocyte nuclear factor‐1*α* was identified in a genetic study as the cause of a new type of antibody negative early onset diabetes (Simaite et al. 2014), but there is to our knowledge no known connection between renal complications and differential expression levels of the native protein. Similarly to PCBD1 the proteins NEDD8, ELOC, FRRS1, GLRX3, and PSAP had lower protein abundances in the liraglutide group and higher abundances in the insulin group, compared to each other and in most cases compared to the db/db vehicle mice. Both NEDD8 and ELOC have previously been shown to be connected to E3 ubiquitin ligase function (den Besten et al. 2012; Andresen et al. 2014), but to the best of our knowledge there are no known function of those proteins in DN.

### Limitations of the study

Our aim was to investigate whether insulin and GLP‐1R agonism have separate effects on the kidney proteome in the db/db mouse model, which we conclude that they have. From an ethical point of view, this conclusion has been reached without the overuse of animals. Nonetheless, this study would have benefitted from a larger cohort, since many proteins of interest, identified as of importance for the mouse group separation in the multivariate analyses, not had significant abundance levels in the univariate statistical tests.

## Conclusion

In summary, this study shows that liraglutide and insulin give rise to distinct abundances of several proteins in the db/db mouse kidney, thereby separating the effects of the two treatments. These proteome differences were not dependent on blood glucose levels or albuminuria in the mice.

## Additional information

### Availability of data and materials

Shotgun submission details:

Project Name: dbdbLiraglutide

Project accession: PXD002988

Project DOI: 10.6019/PXD002988

SRM submission details:

http://www.peptideatlas.org/PASS/PASS00840

Project Name: Lira_Insulin_DN_dbdb

Project accession: PASS00840

## Conflict of Interest

Novo Nordisk markets liraglutide for the treatment of diabetes and obesity. JN and JNM are full‐time employees of Novo Nordisk and hold minor share portions as part of their employment. LL is a former employee of Novo Nordisk and holds a minor share portion.

## Data Accessibility

## Supporting information




**Table S1.** (a) Guide to sample names in Proteome Exchange
**Table S2.** (b) Peptide file from Progenesis software. Included is Uniprot accession number, Mascot score, MS parameters and peptide intensities normalized within Progenesis.
**Table S3.** (c) Uniprot accession number and peptide shotgun MS data used for identification and quantification.
**Table S4.** (d) Additional data of the reported proteins. Mean values and standard deviations (SD) from SRM MS or ^A^shotgun MS. *P*‐values calculated using ANOVA with Tukey *post hoc* test, *P*‐values <0.05 were considered significant, values above 0.05 are shown in grey.
**Table S5.** (e) SRM transition list
**Figure S1.** STRING known and predicted interaction network.
